# An optimized peritonitis-induced ACLF model that reproduces the full spectrum of extrahepatic organ failures in mice

**DOI:** 10.1097/HC9.0000000000000744

**Published:** 2025-06-19

**Authors:** Roger Flores-Costa, Marta Duran-Güell, Berta Romero-Grimaldo, Bryan J. Contreras, Albert Salvatella, María Belén Sánchez-Rodríguez, Frank Uschner, Sabine Klein, Alba Diaz, Estefanía Huergo, David Gómez-Cabrero, Jordi Bosch, Pierre-Emmanuel Rautou, Jonel Trebicka, Cristina López-Vicario, Joan Clària

**Affiliations:** 1 Biochemistry and Molecular Genetics Service, Hospital Clínic—IDIBAPS and CIBERehd, Barcelona, Spain; 2 European Foundation for the Study of Chronic Liver Failure (EF CLIF) and Grifols Chair, Barcelona, Spain; 3 Department of Internal Medicine B, University of Münster, Münster, Germany; 4 Pathology Service, Hospital Clínic—IDIBAPS and CIBERehd, Barcelona, Spain; 5 Translational Bioinformatics Unit, Navarrabiomed, Fundacion Miguel Servet, Universidad Publica de Navarra (UPNA), IdiSNA, Pamplona, Spain; 6 Biological and Environmental Science and Engineering Division, King Abdullah University of Science and Technology (KAUST), Thuwal, Saudi Arabia; 7 Microbiology Service, Hospital Clínic, Barcelona, Spain; 8 Hôpital Beaujon and INSERM, Centre de Recherche sur l’Inflammation, Paris, France; 9 Department of Biomedical Sciences, University of Barcelona, Barcelona, Spain

**Keywords:** decompensated cirrhosis, experimental model, liver, multi-organ failure

## Abstract

**Background:**

Acute-on-chronic liver failure (ACLF), which develops in patients with acutely decompensated cirrhosis, is characterized by multiple extrahepatic organ failures leading to high short-term mortality. Although major advances in the understanding of ACLF have been accomplished in the last years, the understanding of driving mechanisms underlying ACLF is hindered by the lack of proper animal models that faithfully reproduce both the systemic hyperinflammatory response and the full spectra of extrahepatic organ failures present in this condition.

**Methods:**

ACLF was induced by acute induction of polymicrobial peritonitis secondary to the ligation and puncture of the cecum (CLP) in mice with chronic carbon tetrachloride (CCl_4_)-induced cirrhosis. The study included three groups: CCl_4_+CLP (n=10) mice with cirrhosis which underwent CLP surgery; CCl_4_+sham mice (n=10) and control mice (n=10).

**Results:**

As compared to CCl_4_+sham, CCl_4_+CLP mice had higher short-term mortality and exhibited more severe hypoalbuminemia and hyperbilirubinemia, significantly higher AST and GGT levels and higher liver inflammatory burden. CCl_4_+CLP mice also showed increased serum creatinine and BUN levels and up-regulated expression of *Kim-1, Il-6* and *Tnf* in the kidney, lower oxygen saturation (SpO_2_), higher serum renin concentration, higher international normalized ratio (INR) and worse neurological behavior test scores than CCl_4_+sham and control mice. In addition, CCl_4_+CLP mice showed widespread bacterial tissue colonization and exhibited increased serum cytokine levels, which correlated with the intensity of organ impairments.

**Conclusion:**

The CCl_4_+CLP model reproduces the full spectra of extrahepatic organ impairments present in patients with ACLF and represents an optimized murine model to experimentally explore the pathophysiology of this disease as well as new therapeutic approaches.

## Graphical abstract

**Figure:**
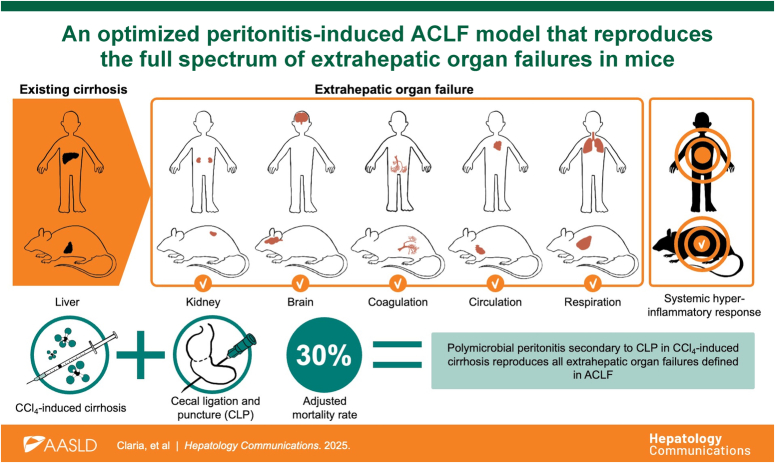

## INTRODUCTION

Acute-on-chronic liver failure (ACLF) frequently develops in patients presenting acute decompensation (AD) of preexisting cirrhosis.^1^,^2^ ACLF is clinically characterized by the concomitant appearance of several extrahepatic organ failures, which result in high short-term mortality.^1^,^2^ At the pathophysiological level, the development of ACLF is closely associated with the presence of intense systemic inflammation and impaired immune defense responses, together with mitochondrial dysfunction and altered blood metabolomic and lipidomic profiles.^3^,^4^,^5^,^6^,^7^ However, a more in-depth analysis of the mechanisms underlying ACLF at the tissue and organ level is needed to fully understand the pathophysiology of this syndrome. Unfortunately, direct access to tissues and organs from critically ill patients with ACLF represents a manifest limitation.

Experimental models offer the possibility to overcome the patient’s limitation of performing factual mechanistic studies in tissues and organs. Up to now, several attempts have been made to model ACLF in rodents, including the induction of cirrhosis by carbon tetrachloride (CCl_4_) or bile duct ligation (BDL) combined with acute insults, such as lipopolysaccharide (LPS), acetaminophen, double dose of CCl_4,_ i.p. injection of bacteria or single alcohol binge.^8^,^9^,^10^,^11^,^12^,^13^ Unfortunately, most of these models are limited to reproducing only one extrahepatic organ failure apart from the liver and fall short in replicating the whole spectrum of organ failures defined in ACLF. Therefore, the development of novel models that fully epitomize the clinical characteristics of ACLF represents an urgent unmet need.

Here we describe an optimized murine model of ACLF able to reproduce the systemic hyperinflammatory condition, the hepatic and extrahepatic organ failures, and the reduced short-term survival associated with ACLF. This model is based on the induction of polymicrobial peritonitis, one of the most frequent causes of hospitalization in patients with decompensated cirrhosis,^14^ through the ligation and puncture of the cecum in mice with preexisting CCl_4_-induced cirrhosis. By modifying the proximal distance of the ligation to the ileocecal valve and by using different gauge needles to puncture the cecum,^15^ the cecal ligation and puncture (CLP) model allows for fine-tuning the severity of organ impairments and therefore to capture the existing heterogeneity of this disease. Of note, we developed this model in mice to enable the use of genetically engineered animals and further facilitate mechanistic studies aimed at understanding the pathophysiology of ACLF and to facilitate the development and testing of new therapies.

## METHODS

### Experimental design

Male C57BL/6J mice (Charles River Laboratories) were housed following the ARRIVE guidelines (https://nc3rs.org.uk/arrive-guidelines). Briefly, mice were housed in conventional cages on woodchip bedding provided with environmental enrichments such as bedding material, chew implements, and hiding structures. A maximum of 5 animals were allocated in each cage, placed in a 20–25 °C temperature-controlled non-SPF facility with 50%–60% humidity and a 12-hour light/dark cycle and given ad libitum access to food and water. No animals were excluded from the study for manifesting poor shape or bad conditioning. Starting at 6 weeks of age [20–25 g body weight (b.w.)], 20 mice received i.p. injections of CCl_4_ (1 µL/g b.w.) diluted in olive oil 2 times per week for at least 12 weeks. A control group of 10 mice receiving vehicle (olive oil) was also included. Body weight was regularly assessed and once the mice receiving CCl_4_ had signs of ascites in the peritoneal cavity (Supplemental Figure S1, http://links.lww.com/HC9/C23), as detected by palpation of the abdomen manifesting a doughy, almost fluctuant sensation, they were randomly divided into a group that was sham operated (CCl_4_+sham, n=10) and another group that underwent CLP surgery (CCl_4_+CLP, n=10). A group of 5 non-cirrhotic mice that underwent CLP was also included. For the CLP procedure, mice were anesthetized with inhaled isoflurane/oxygen and were subcutaneously administered buprenorphine (0.3 mg/kg) before performing laparotomy to isolate the cecum. We performed preliminary experiments using different gauge needles to puncture the cecum and modifying the distance of the ligation to the ileocecal valve to adapt the mortality rate in our model to that observed in human disease, which is about 30%. To achieve this goal, ~2/3 of the cecum was ligated with a 3–0 silk suture and punctured with a 30-gauge needle. Sham-operated mice underwent laparotomy without ligation and puncture of the cecum. Thereafter, the abdominal wall and skin were sutured with 3–0 silk and animals were continuously monitored until recovery from anesthesia. Buprenorphine (0.3 mg/kg) was administered every 12 hours until sacrifice, which was performed by anesthesia overdosage (0.1 mg ketamine/g b.w. and 0.01 mg xylazine/g b.w.) 24 hours after surgery. Prior to sacrifice, neurological behavior tests (NBTs) were performed, and oxygen saturation (SpO_2_) was measured in the tail with a veterinary pulse oximeter (PO-600VET; Pulox). A drop of blood from the tail vein was also collected for measurement of the international normalized ratio (INR) using a Coaguchek XS System (Roche). Immediately after, blood was collected by cardiac puncture and serum allowed to clot before centrifugation at 800*g* for 10 minutes. Finally, liver, epididymal white adipose tissue (eWAT), interscapular brown adipose tissue (iBAT), kidneys, lungs, heart, brain, and spleen were excised and rinsed in DPBS^++^. Then, portions of liver, kidney, and lung were fixed in 10% formalin and embedded in paraffin, and the remaining tissues were snap-frozen in liquid nitrogen for further analysis. All studies were conducted in accordance with the criteria of the Investigation and Ethics Committee of the Universitat de Barcelona and the European Community laws governing the use of experimental animals.

### Biochemical analyses

Serum concentrations of glucose, total cholesterol, inorganic phosphorus, calcium, lactate dehydrogenase (LDH), ALP, bilirubin, ALT, AST, GGT, creatinine, and blood urea nitrogen (BUN) were determined using the Dri-Chem NX600 equipment (Fujifilm).

### Serum albumin and renin levels

Serum albumin and renin levels were assessed using mouse albumin and renin ELISA kits (ab108791; Abcam and Thermo Fisher Scientific, respectively), following the manufacturer’s instructions.

### Histological analysis of tissue injury

Paraffin-embedded liver, kidney, and lung tissues were cut into 5-μm sections and stained with hematoxylin–eosin at the IDIBAPS Biobank. Necroinflammatory injury was analyzed at 200× magnification in a blinded fashion by a registered pathologist (Alba Diaz). Inflammation was scored by analyzing the number of inflammatory foci/field. For assessment of liver cirrhosis, sections were incubated for 10 minutes in 0.5% thiosemicarbazide and stained in 0.1% Sirius red F3B in saturated picric acid for 1 hour, followed by a wash with a 0.5% acetic acid solution. Liver sections were visualized at 40× and 200× magnifications in a Nikon Eclipse E600 microscope and scored by the extent and disposition of collagen fibers by the registered pathologist.

### Transmission electron microscopy (TEM)

Livers were fixed in 2% paraformaldehyde and 2.5% glutaraldehyde, post-fixed with 1% osmium tetroxide and 0.8% potassium ferrocyanide, dehydrated in acetone and embedded in Spurr epoxy resin. Four ultrathin sections were obtained from each sample. The ultrathin sections were poststained with uranyl acetate and lead citrate and examined under a JEOL J1010 TEM (Akishima). At least 10 fields per sample were assessed, and images were taken at 15,000×.

### Analysis of serum cytokine levels

Serum levels of granulocyte colony-stimulating factor (G-CSF), granulocyte-macrophage colony stimulating factor (GM-CSF), eotaxin, IL-1β, IL-1L-L-α, IL-4, IL-6, the mouse IL-8 homolog keratinocyte-derived chemokine (KC), IL-10, IL-17, interferon (IFN)-γ-induced protein (IP)-10, monocyte chemoattractant protein-1 (MCP-1), macrophage inflammatory protein (MIP)-1α, tumor necrosis factor-alpha (TNFα), and VEGF were assessed in a MAGPIX system (Luminex Corp.) using a custom-made Milliplex Mouse Expanded Cytokine Magnetic MCYTOMAG-70K (Merck Millipore). Briefly, 25 μL of serum and 25 μL of diluent were added to each well before the addition of premixed microbeads (25 μL). The plate was incubated overnight at 4 °C with shaking, then washed and incubated again with 25 μL of detection antibody for 1 hour. After washing, the plate was incubated with 25 μL of streptavidin–phycoerythrin for 30 minutes, washed twice, and the beads resuspended with 150 μL of sheath fluid and finally analyzed in a MAGPIX system using the Belysa Analysis Software 1.1 (Merck Millipore). Readouts were detected as mean fluorescence intensity by the instrument, and values were subsequently converted to pg/mL by extrapolation from a set of standards that were run simultaneously in the assay.

### Microbiology assessments

Peritoneal fluid smears and liver, kidney, and lung biopsies were collected in thioglycolate medium and immediately cultured for subsequent assessment of the colonizing microorganisms at the Microbiology Service of the Hospital Clínic of Barcelona.

### Gene expression analysis by real-time PCR

Isolation of total RNA was performed using the TRIzol reagent (MRC). RNA concentration was assessed in a NanoDrop-1000 spectrophotometer (NanoDrop Technologies), and its integrity was tested on a 6000 LabChip in a Bioanalyzer 2100 (Agilent Technologies). cDNA synthesis from 1000 ng of total RNA was performed using the High-Capacity cDNA Archive Kit (Applied Biosystems). For real-time PCR quantification, validated and pre-designed TaqMan Gene Expression Assays were used: *Il1b* (ID: Mm00434228_m1), *Il6* (ID: Mm00446190_m1), *Tnf* (ID: Mm00443258_m1), and kidney injury molecule-1 (*Kim1,* also known as *Havcr1*; ID: Mm00506686_m1), using β-actin (*Actb*; ID: Mm00607939_s1) as endogenous control. Real-time PCR amplifications were performed in a 7900HT Fast system (Applied Biosystems). PCR results were analyzed with Sequence Detector Software version 2.3 (Applied Biosystems). The amount of target gene, normalized to β-actin and relative to a calibrator, was determined by the arithmetic equation 2^−ΔΔCt^ described in the comparative Ct method.

### Assessment of brain impairment

The NBT assessment was performed based on the revised version of the neurobehavioral severity scale for rodents.^16^ A total of 10 tests were performed, including general balance test, tail raise test, landing test, drag test, righting reflex, ear reflex, eye reflex, sound reflex, tail reflex, and paw flexion reflex. In addition, the brain water content (BWC) was measured by excising the frontal part of the brain (~5 mm^3^) and weighing it on an electronic scale to obtain the brain wet weight. Thereafter, samples were heated in an oven at 100 °C for 24 hours to obtain the brain dry weight.^17^,^18^ The BWC was calculated using the following equation: BWC (%) = (Brain Wet Weight−Brain Dry Wet/Brain Wet Weight)×100.

### Organ impairment criteria

To discern the degree of impairment of the different organ systems, thresholds of normality based on established reference intervals were set for each marker of organ injury. For liver, serum bilirubin levels between 0.2 and 0.6 mg/dL were considered normal, while values above 0.6 mg/dL were considered to reflect liver impairment.^19^ For the kidney, creatinine values in the range of 0.2–0.5 mg/dL were close to normality, while impairment was considered with serum creatinine levels above 0.5 mg/dL.^19^ For the brain, animals with BWC >80% or with a NBT score higher than 4 were considered to suffer from brain impairment.^16^,^17^,^18^,^19^,^20^ Coagulation and circulation impairments were defined as INR values >1.2 and serum renin concentration higher than 1 µg/mL, respectively.^21^,^22^,^23^,^24^ Respiratory impairment was considered in mice exhibiting SpO_2_ values lower than 70%.^25^,^26^

### Data and statistical analysis

Statistical analysis was performed with GraphPad Prism version 8.0. For single comparisons, the Student unpaired *t* test was used. For multiple comparisons, an ANOVA of the data in which a significant (*p*<0.05) main effect followed by Dunnett post hoc test was performed. The results are presented as mean±SEM or median with IQR, depending on data distribution. All measurements were undertaken in at least 2 technical replicates. Associations between serum cytokines and markers of organ impairment were analyzed by Spearman correlation. For the analysis of cytokine results, values below the limit of quantification (LOQ) were imputed by using a 0.25×LOQ. For principal component analysis (PCA) and heatmap representations, data were log transformed, centered (subtracting each parameter's mean from the corresponding parameter's individual values) and scaled (dividing the values by their corresponding SD). Spearman’s correlation, PCA analyses, and heatmap graphs were performed using R v4.1.1 statistical software.

All authors had access to the study data and had reviewed and approved the final manuscript.

## RESULTS

### Body and adipose tissue weight and serum biochemistry

**FIGURE 1:**
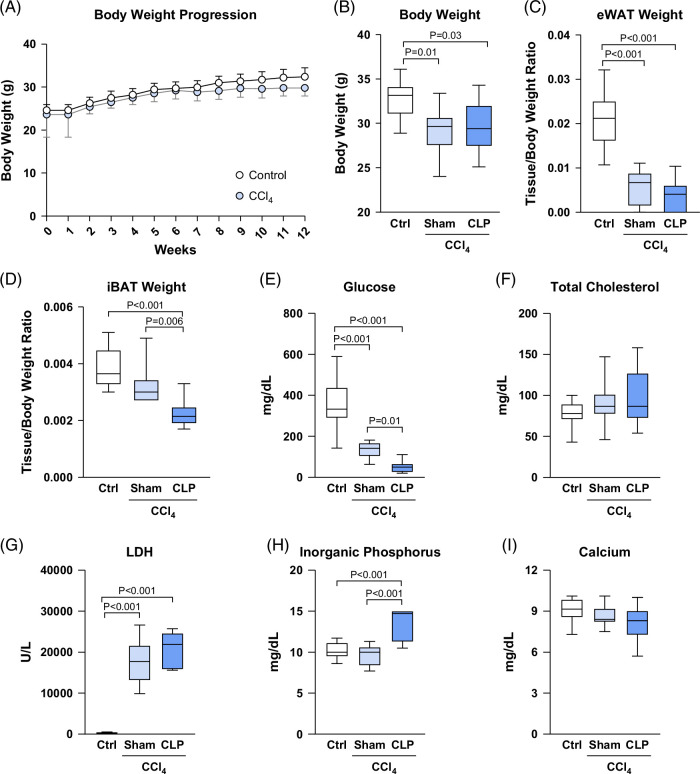
Changes in body and adipose tissue weight and serum biochemistry in mice with CCl_4_-induced cirrhosis undergoing CLP. (A) Body weight progression during the 12 weeks of treatment in control mice (n=10) and CCl_4_-induced cirrhotic mice (n=20). (B) End-point body weight, (C) epididymal white adipose tissue (eWAT), and (D) interscapular brown adipose tissue (iBAT) in control mice (n=10) and CCl_4_-induced cirrhotic mice (n=20). (E–I) Serum glucose, total cholesterol, lactate dehydrogenase (LDH), inorganic phosphorus, and calcium levels. Results from body weight progression are expressed as mean±SEM. Results are represented in box plots; the boxes show the interquartile range, the median values (horizontal lines), and the bars denote the highest and lowest values of the distribution. Abbreviations: CCl_4_, chronic carbon tetrachloride; CLP, cecal ligation and puncture; eWAT, epididymal white adipose tissue; iBAT, interscapular brown adipose tissue; LDH, lactate dehydrogenase.

During the 12 weeks of CCl_4_ administration, body weight remained unchanged in comparison to the control group (Figure 1A). However, CCl_4_-treated mice experienced significant weight loss after CLP or sham surgery (Figure 1B). This postoperative change was associated with a reduction in eWAT in both groups, whereas iBAT was only significantly affected in the CCl_4_+CLP group (Figures 1C, D). In addition, lower levels of serum glucose were observed postoperatively in both groups, although this effect was more pronounced in the CLP group (Figure 1E). No changes in serum cholesterol were observed across the 3 study groups (Figure 1F). LDH levels were increased in both the CLP and sham groups (Figure 1G), while inorganic phosphorus concentrations were only elevated in CCl_4_+CLP mice (Figure 1H). No changes in plasma calcium levels were observed (Figure 1I).

### Changes in liver parameters in mice with cirrhosis that underwent CLP

**FIGURE 2:**
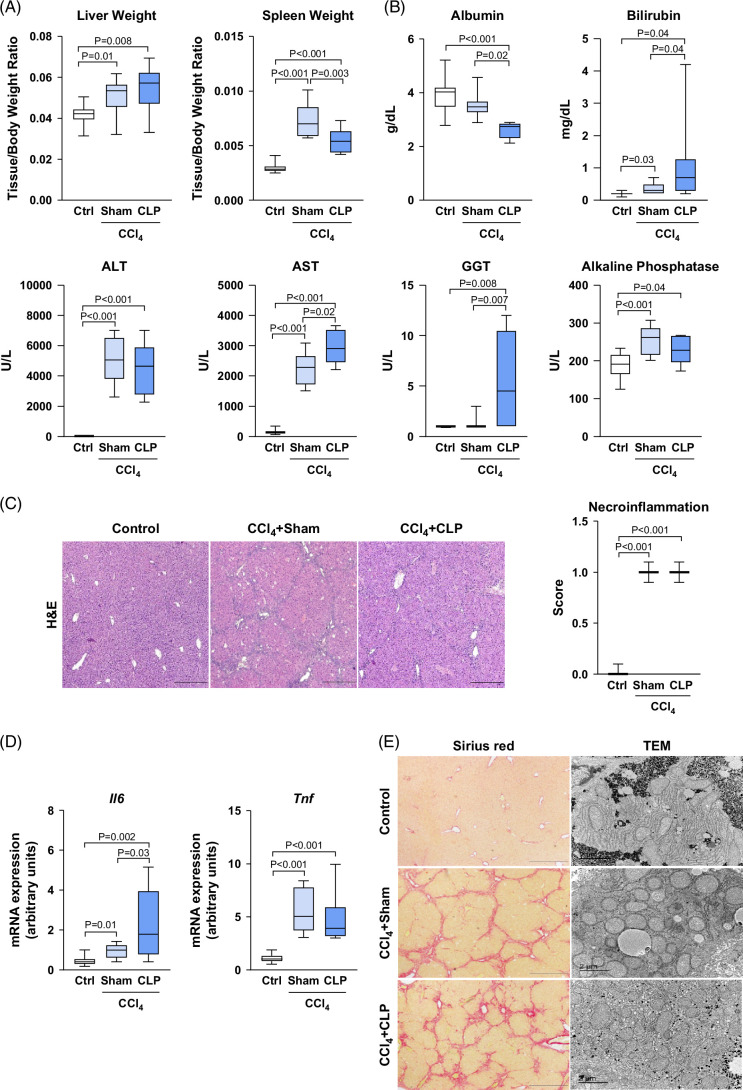
Liver changes after CLP in mice with CCl_4_-induced cirrhosis. (A) Liver and spleen weight, expressed as tissue-to-body weight ratio and serum albumin. (B) Albumin, bilirubin, ALT, AST, GGT, and ALP levels in mice from control (n=10), CCl_4_+sham (n=10), and CCl_4_+CLP (n=10) groups. (C) Representative photomicrographs (40× magnification) of liver sections stained with H&E. Necroinflammation score was calculated by a registered pathologist by analyzing the H&E-stained sections. (D) Hepatic *Il6* and *Tnf* gene expression determined by real-time PCR. (E) Representative photomicrographs (40× magnification) of liver sections stained with Sirius red. Scale bar=500 μm (left panel) and representative photomicrographs (15,000× magnification) of liver sections obtained in the TEM. Scale bar=2 μm (right panel). Results are represented in box plots; the boxes show the interquartile range, the median values (horizontal lines), and the bars denote the highest and lowest values of the distribution. Abbreviations: CCl_4_, chronic carbon tetrachloride; CLP, cecal ligation and puncture; H&E, hematoxylin and eosin; TEM, transmission electron microscopy.

Mice with CCl_4_-induced cirrhosis exhibited higher liver and spleen to body weight ratios, although the latter was significantly less pronounced in those that underwent CLP (Figure 2A). Hypoalbuminemia, hyperbilirubinemia, and increased AST and GGT levels were more severe in CCl_4_+CLP than in the sham group, with similar elevations in ALT and ALP (Figure 2B). CCl_4_-treated mice showed increased liver necroinflammatory injury and expression of inflammatory cytokines compared to the control group, with no differences between CCl_4_+CLP and CCl_4_+sham mice, except for *Il6*, which showed higher upregulation in the CCl_4_+CLP group (Figures 2C, D). Compared to controls, both groups of CCl_4_-treated mice showed a similar extent of Sirius red staining and similar damage in the ultrastructure morphology of the liver parenchyma, characterized by smaller and more round-shaped mitochondria (Figure 2E).

### Changes in renal parameters in mice with cirrhosis that underwent CLP

**FIGURE 3:**
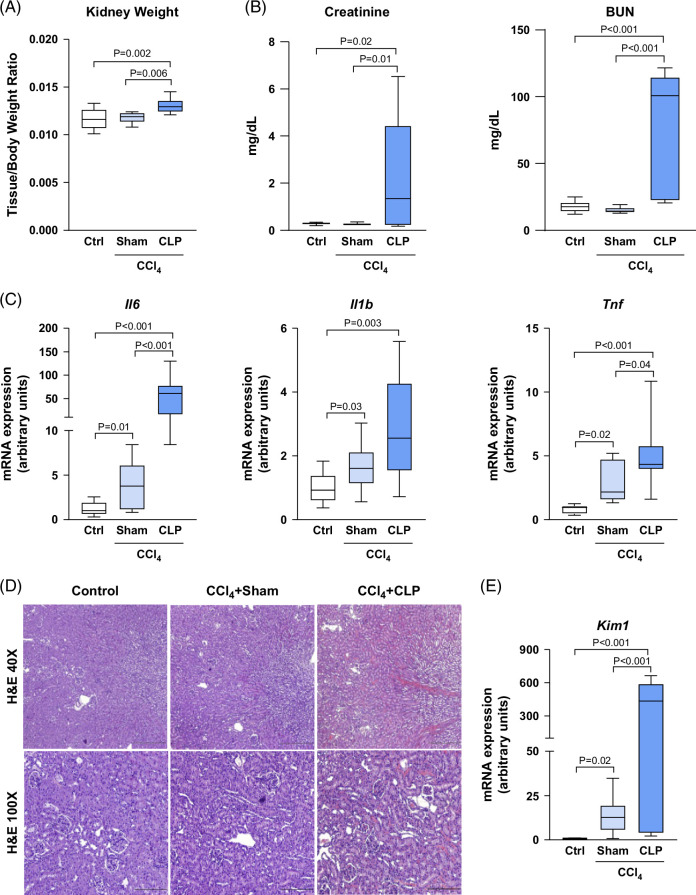
Renal changes after CLP in mice with CCl_4_-induced cirrhosis. (A) Kidney weight expressed as tissue-to-body weight ratio. (B) Serum creatinine and BUN levels. (C) *Il6, Il1b*, and *Tnf* expression in renal tissue as determined by real-time PCR. (D) Representative photomicrographs (40× and 100× magnifications) of kidney sections stained with H&E. (E) *Kim1* expression in the kidney as determined by real-time PCR. Results are represented in box plots; the boxes show the interquartile range, the median values (horizontal lines), and the bars denote the highest and lowest values of the distribution. Abbreviations: BUN, blood urea nitrogen; CCl_4_, chronic carbon tetrachloride; CLP, cecal ligation and puncture; H&E, hematoxylin and eosin.

We next scrutinized the CCl_4_+CLP model for the most common extrahepatic organ failure in ACLF.^27^ Mice induced to cirrhosis by CCl_4_ did not exhibit significant changes in kidney-to-body weight ratio or in functional markers of renal impairment such as serum creatinine and BUN (Figures 3A, B). However, these mice showed increased kidney-to-body weight ratio and significantly higher serum creatinine and BUN levels after CLP in comparison to both control and CCl_4_+sham mice (Figures 3A, B). CCl_4_+CLP mice also showed significant upregulation of the inflammatory markers *Il6* and *Tnf* (Figure 3C). Furthermore, histological assessment of the kidneys revealed signs of acute tubular necrosis in about 30% of mice of the CCl_4_+CLP group (Figure 3D). Consistent with this, the expression of *Kim1*, a specific gene marker for tubular kidney injury, was upregulated in CCl_4_+CLP compared to both control and CCl_4_+sham groups (Figure 3E).

### Changes in respiratory and cerebral functions and coagulation, and circulatory systems in mice with cirrhosis that underwent CLP

**FIGURE 4:**
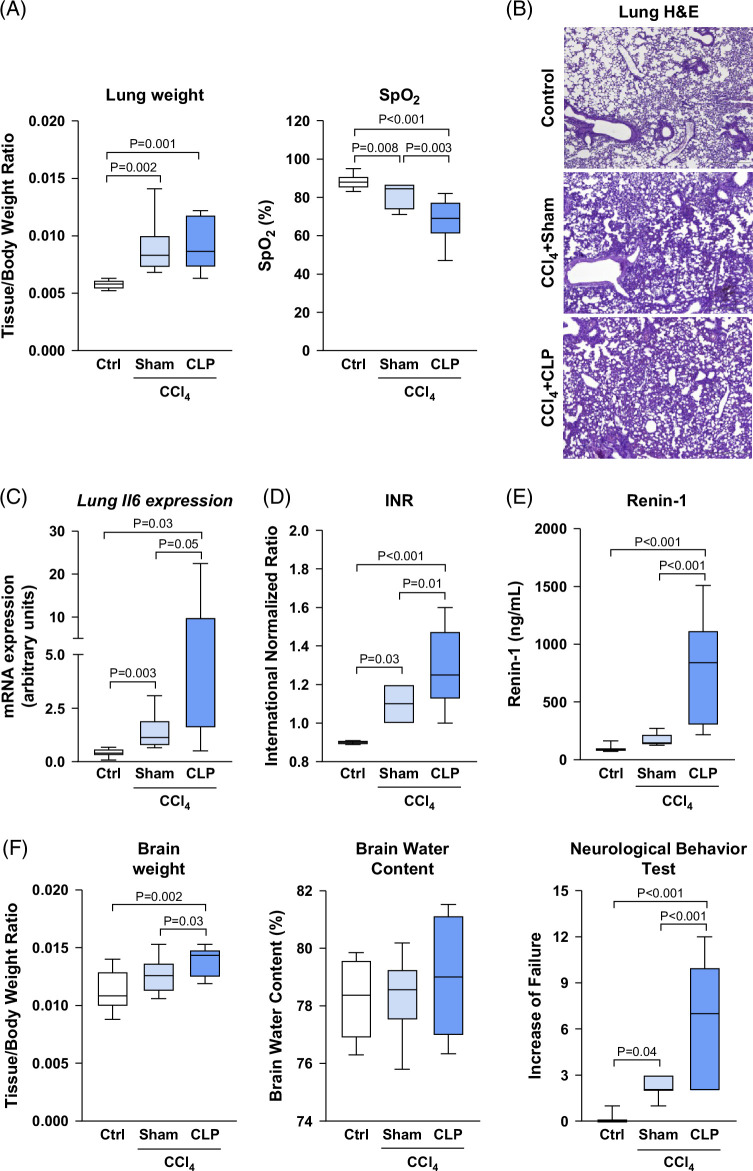
Changes in lung and brain function and in circulatory and coagulation systems after CLP in mice with CCl_4_-induced cirrhosis. (A) Pulmonary parameters, namely lung weight expressed as tissue-to-body weight ratio and oxygen saturation expressed as SpO_2_, were measured in the animals prior to sacrifice. (B) Representative photomicrographs (40× magnification) of lung sections stained with H&E. (C) Expression of *Il6* in the lung as determined by real-time PCR. (D) INR was measured in the mice prior to sacrifice. (E) Serum renin-1 concentration. (F) Brain parameters, namely brain weight expressed as tissue-to-body weight ratio, brain water content expressed as percentage of water in the tissue (see the Methods section), and neurological behavior test were performed and scored as the increase of failure in all the mice of the study (see the Methods section). Results are represented in box plots; the boxes show the interquartile range, the median values (horizontal lines), and the bars denote the highest and lowest values of the distribution. Abbreviations: CCl_4_, chronic carbon tetrachloride; CLP, cecal ligation and puncture; H&E, hematoxylin and eosin; INR, international normalized ratio.

We next examined other frequent extrahepatic organ system failures in mice with CCl_4_-induced cirrhosis that underwent CLP surgery. Both CCl_4_+sham and CCl_4_+CLP mice showed increased lung-to-body weight ratio and reduced SpO_2_ levels compared to the control group, although the SpO_2_ reduction was significantly more pronounced in CCl_4_+CLP than in CCl_4_+sham mice (Figure 4A). CCl_4_+CLP mice also showed higher lung inflammatory injury as revealed by hematoxylin–eosin staining and increased *Il6* expression in comparison to CCl_4_+sham mice (Figures 4B, C). CCl_4_+CLP mice also showed significantly higher INR and serum renin-1 levels than CCl_4_+sham and control mice (Figures 4D, E), indicating that, in addition to liver and respiratory failure, this ACLF model also develops coagulation and circulatory failure. Finally, we tested the status of cerebral function by assessing brain weight, BWC, and NBT. Figure 4F shows that CCl_4_+CLP mice had increased brain-to-body weight ratio, no changes in BWC, but significantly higher NBT scores indicative of neurological behavior failure.

### Systemic hyperinflammation in mice with cirrhosis that underwent CLP

**FIGURE 5:**
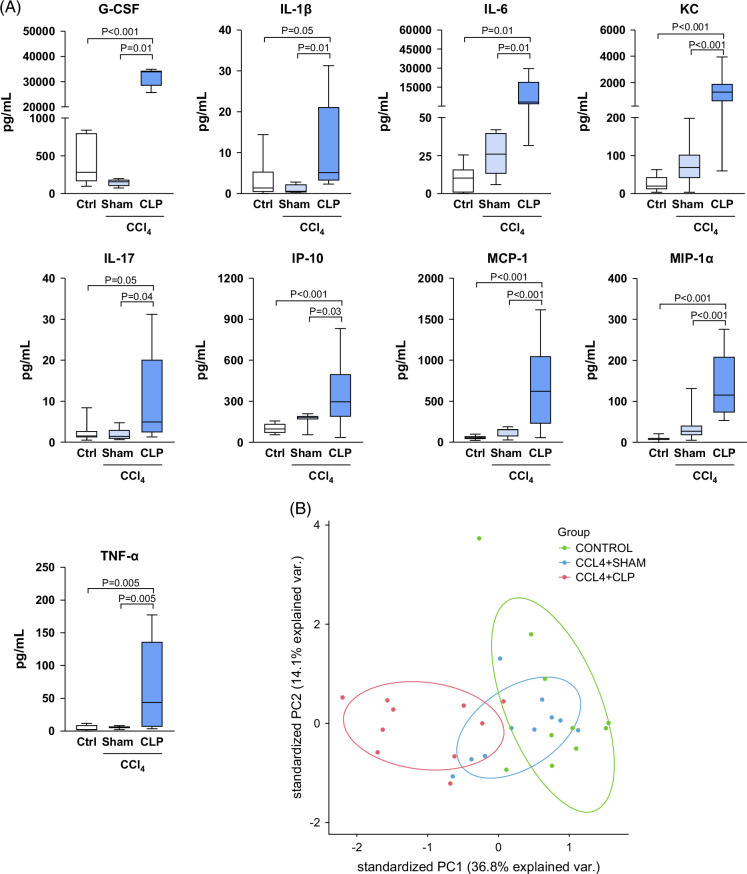
Exacerbated systemic inflammation in mice with CCl_4_-induced cirrhosis undergoing CLP. (A) Serum G-CSF, IL-1β, IL-6, IL-8 (KC), IL-17, IP-10, MCP-1, MIP-1α, and TNF-α protein levels were determined by Milliplex technology. (B) Keratinocyte-derived chemokine (PCA) displays data from cytokines in all the animals from the study. Results are expressed as box plots, the boxes show the interquartile range, the median values (horizontal lines), and the bars denote the highest and lowest values of the distribution. Abbreviations: CCl_4_, chronic carbon tetrachloride; CLP, cecal ligation and puncture; G-CSF, granulocyte colony-stimulating factor; IP-10, interferon (IFN)-γ-induced protein 10; KC, keratinocyte-derived chemokine; MCP-1α, monocyte chemoattractant protein-1; MIP-1, macrophage inflammatory protein-1; TNF-α, tumor necrosis factor-α.

Exuberant systemic inflammation is a hallmark of ACLF.^3^ To determine whether this is also characteristic of mice with CCl_4_-induced cirrhosis that underwent CLP, serum cytokine and chemokine levels were measured in CCl_4_+CLP mice and compared to those of CCl_4_+sham and control mice. Among the 16 cytokines measured, GM-CSF and VEGF were not detected in the circulation of any mouse under any experimental condition. Compared to CCl_4_+sham and control animals, mice with CCl_4_+CLP showed significantly higher levels of G-CSF, IL-1β, IL-6, and KC (the mouse IL-8 homolog), IL-17, IP-10, MCP-1, MIP-1α, and TNF-α (Figure 5A). No differences were observed in the serum levels of IL-1α, IFN-γ, eotaxin, IL-4, and IL-10 (Supplemental Figure S2, http://links.lww.com/HC9/C23). Of interest, when we used PCA to reduce the dimension of the cytokine–chemokine data, we obtained a clear separation of the CCl_4_+CLP group from the CCl_4_+sham and control groups (Figure 5B).

**FIGURE 6:**
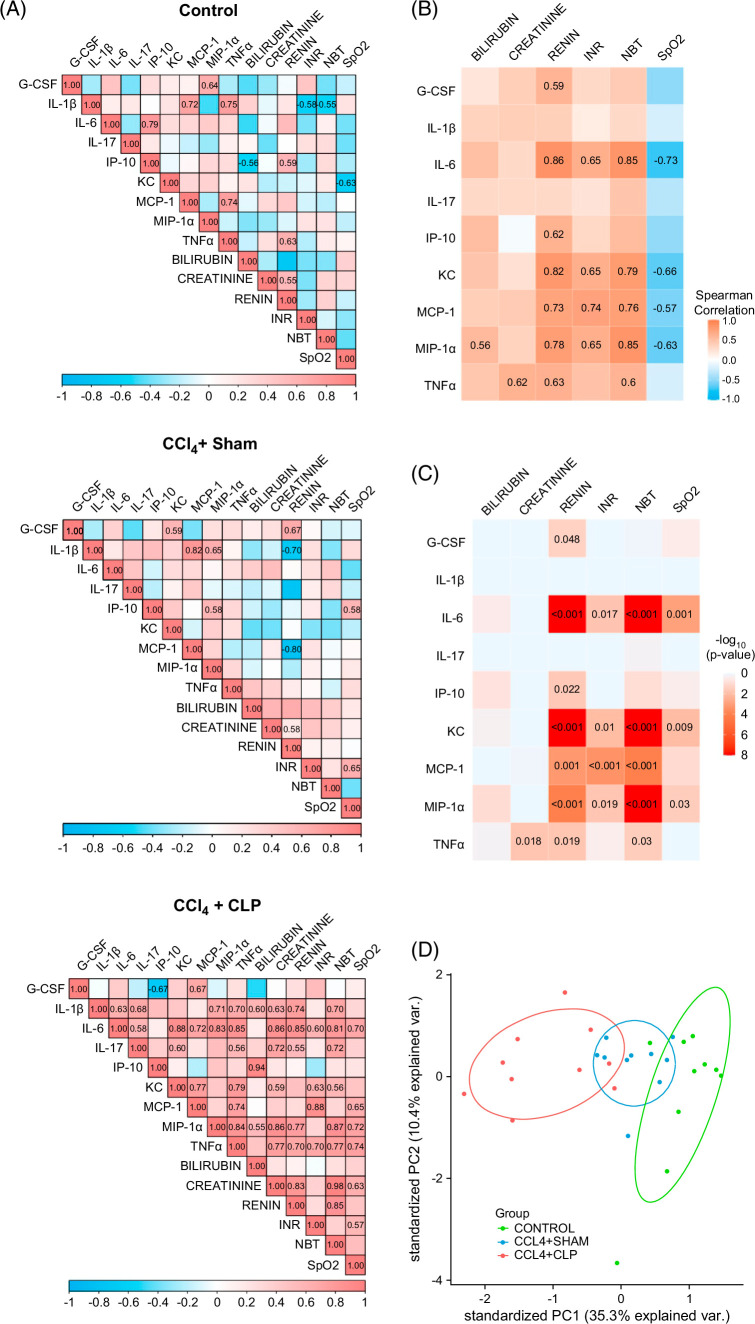
Correlation of inflammatory markers with organ impairments. (A) Correlation plots showing the Spearman correlation between the serum cytokines and the parameters used to define the organ failures in control (n=10), CCl_4_+sham (n=10), and CCl_4_+CLP (n=10). (B) Heatmap showing the strength of Spearman correlation between the serum cytokines and the parameters used to define the organ failures in all the mice from the study. (C) Heatmap showing the statistical significance of Spearman correlation between the serum cytokines and the parameters used to define the organ failures in all the mice from the study. (D) PCA displaying data from cytokines and parameters used to define the organ failures [liver: bilirubin, kidney: creatinine, circulation: serum renin concentration, coagulation: INR, brain: neurological behavior test, and respiration: oxygen saturation (SpO_2_)] in all the animals from the study. Abbreviations: CCl_4_, chronic carbon tetrachloride; CLP, cecal ligation and puncture; INR, international normalized ratio; NBT, neurological behavior test; PCA, principal component analysis.

We next explored plausible correlations between serum cytokine–chemokine levels and markers of organ impairment (ie, bilirubin, creatinine, NBT, INR, renin, and SpO_2_). This analysis identified a stepwise increase in the degree of correlation between cytokines–chemokines and organ failures, being low to medium in control and CCl_4_+sham mice, respectively, and strong in the CCl_4_+CLP group (Figure 6A). The close connection between the increased cytokine levels and the manifestation of organ failures can be better appreciated by pooling the data independently of the study group and plotting the heatmaps of the strength of the correlations (Figure 6B) and their statistical significance (Figure 6C). Importantly, the combination of cytokine–chemokines and markers of organ failure allowed a better PCA separation of CCl_4_+CLP mice from CCl_4_+sham and control mice (Figure 6D).

### Categorization of organ impairment, bacterial colonization, and survival rates in mice with cirrhosis that underwent CLP

**FIGURE 7:**
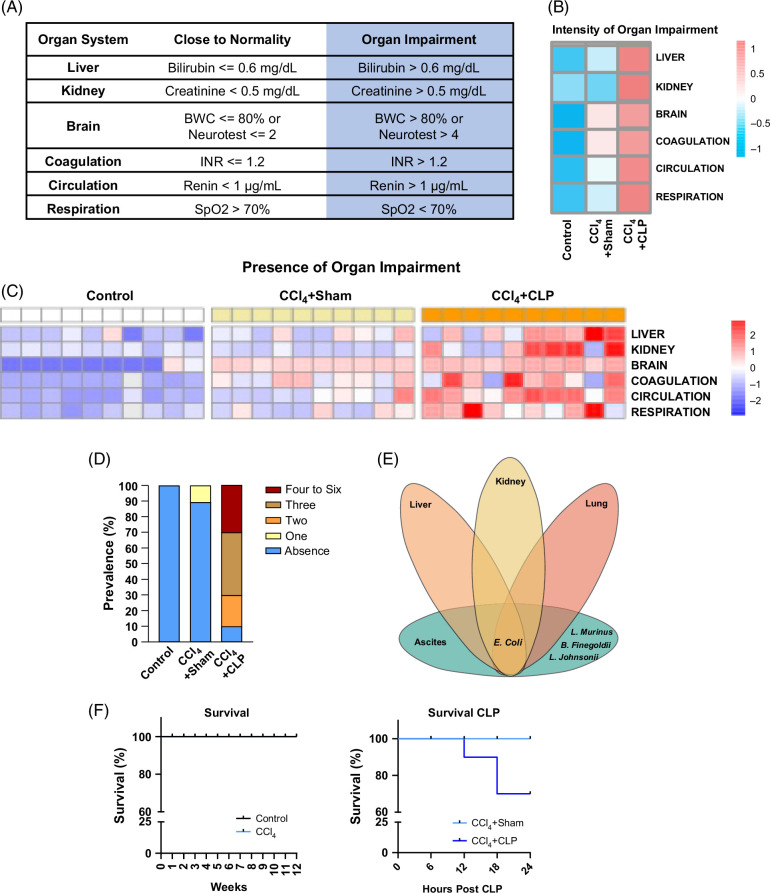
Prevalence of organ impairment, bacterial colonization, and survival rates in mice with CCl_4_-induced cirrhosis undergoing CLP. (A) Adapted definition of organ impairments according to the criteria described in the Methods section. (B) Heatmap of the intensity of organ impairments in control, CCl_4_+sham, and CCl_4_+CLP mice, calculated from the mean values of the scores using the criteria defined in panel A. (C) Heatmap of the prevalence of organ impairment in each animal, using the criteria described above. (D) A stacked column graph showing the prevalence of the number of organ impairments in control, cirrhotic, and cirrhotic mice undergoing CLP. Results are expressed as a percentage of the total. (E) Venn diagram displaying the microorganisms detected in peritoneal fluid and hepatic, renal, and pulmonary tissues from CCl_4_+CLP mice. (F) Survival of mice induced to cirrhosis by CCl_4_ administration (n=20) and control mice (n=10), and 24-hour survival of sham-operated cirrhotic mice (n=10) and cirrhotic mice undergoing CLP surgery (n=10). Abbreviations: BWC, brain water content; CCl_4_, chronic carbon tetrachloride; CLP, cecal ligation and puncture; INR, international normalized ratio.

To facilitate the translation of the results from the CCl_4_+CLP mouse model to the clinical scenario, we categorized the organ impairments present in this model by resembling most of the clinical and laboratory parameters included in the modified sequential organ failure assessment CLIF-C score developed for ACLF patients.^2^,^28^ Figure 7A describes the functionality of the liver, kidney, and brain and the coagulation, circulatory, and respiratory systems based on the literature and mouse physiological databases.^16^,^17^,^18^,^19^,^20^,^21^,^22^,^23^,^24^,^25^,^26^ We then plotted the numeric values for each marker of organ impairment in a heatmap (Figure 7B), which revealed that organ impairments peaked in CCl_4_+CLP mice. The percentage of animals affected by each individual organ impairment is detailed in Supplemental Table S1, http://links.lww.com/HC9/C23, which confirms that the CCl_4_+CLP group had a greater incidence of all impairments. In addition, the individual data for each animal and for each group are represented in Figure 7C, indicating that this model also reproduces the existing patients’ heterogeneity. This heterogeneity can be better visualized by distributing the mice according to the number of organ impairments, which confirmed the high variability (ie, from 1 to 3 or more organ impairments) existing in CCl_4_+CLP mice (Figure 7D). Interestingly, we detected bacterial colonization with *Escherichia coli, Lactobacillus murinus*, *Lactobacillus Johnsonii*, and *Bacteroides finegoldii* in the peritoneal fluid and *Escherichia coli* in liver, kidney, and lung tissues in CCl_4_+CLP mice but not in the other 2 groups (Figure 7E). We observed that while survival at 24 hours was not compromised in mice with CCl_4_-induced cirrhosis after 12 weeks of CCl_4_ treatment, survival in the CCl_4_+CLP group was 70% (Figure 7F), a survival rate comparable to the short-term survival of patients with ACLF.^2^ Finally, to investigate the pathological changes due to sepsis alone and whether these are exacerbated by the superimposed effects of cirrhosis, we investigated organ function in a non-cirrhotic CLP group of mice. These animals did not show increased liver weight nor liver failure, although they showed signs of liver injury (ie, increased ALT and AST levels), renal failure (ie, increased creatinine levels), and higher INR (indicative of coagulation failure) without evidence of brain failure (Supplemental Figure S3, http://links.lww.com/HC9/C23).

## DISCUSSION

There is an urgent need to have experimental models that closely reproduce organ system failures manifested by patients with ACLF. This requirement stems from the limited access to organs, tissues, and samples from patients, making it very difficult to precisely identify the pathophysiological mechanisms and drivers that lead to the development of ACLF. In addition, such a model is essential to have the possibility to develop and test new therapies that require factual validation in preclinical models. The present study describes a new experimental model based on combining the commonly available model of cirrhosis induction by CCl_4_ administration with the CLP model of polymicrobial-induced peritonitis in mice, to yield a murine model that closely reproduces both the hepatic and extrahepatic organ system failures as well as the systemic hyperinflammatory condition associated with ACLF. Considering that infections in general, and peritonitis in particular, are the most frequent precipitants of ACLF in patients with AD cirrhosis,^14^ the CCl_4_+CLP model appears as an optimal experimental model for investigating the mechanisms and drivers underlying ACLF development.

The CCl_4_+CLP model is new in the sense that it not only reproduces liver failure but also faithfully reproduces all the extrahepatic organ and system *impairments* associated with ACLF. Indeed, the CCl_4_+CLP model concurrently displays impairment in liver, renal, respiratory, cerebral, circulatory and coagulation organ systems, and shows key features of ACLF development such as systemic hyperinflammation and bacterial colonization of peripheral tissues. In addition, the CCl_4_+CLP model shows reduced splenic mass, a phenomenon previously reported by Frydrych et al^29^ using the CLP procedure in obese diabetic mice, which was interpreted by these authors as a dysfunctional emergency granulopoiesis response after acute infection induced by CLP. It is worth noting that several experimental models in both mice and rats have previously been described to investigate the pathophysiology of ACLF. For instance, animals with CCl_4_-induced or BDL-induced cirrhosis have been challenged with LPS, acetaminophen, living bacteria, D-galactosamine, or alcohol binge.^8^,^9^,^10^,^11^,^12^,^13^,^30^ Although these experimental models exhibit liver and/or renal failure, they do not reproduce other extrahepatic organ impairments common in patients with ACLF, such as impaired coagulation, respiratory and circulatory functions, or brain failure. Of interest, performing the CLP procedure in the setting of liver cirrhosis offers some specificities to the CLP model. For example, in contrast to that seen in the CCl_4_-CLP model, induction of CLP in non-cirrhotic healthy mice does not induce respiratory failure, as oxygen saturation was reported unchanged in these animals.^31^ Moreover, in contrast to the CCl_4_-CLP model, the CLP procedure in non-cirrhotic mice does not produce brain and liver failure. Overall, the CCl_4_+CLP model represents a major refinement of the currently existing experimental models and an important advancement in the field.

Our study translated the experimental model to the real clinical situation by adapting the criteria that define organ impairments in patients with ACLF to the CCl_4_+CLP model. According to the EASL–CLIF consortium, ACLF is classified by severity in 3 groups (ACLF-1, 2, and 3) depending on the number of organ failures, which are associated with higher (ACLF-3) or lower (ACLF-1) probability of death at 90 days.^1^,^2^ The need for a definition for organ failures in ACLF patients was met by designing a table assigning scores from 1 to 3 points for the variables associated with liver, kidney, brain, coagulation, and circulatory and respiratory organ system dysfunctions and failures.^1^,^2^ In our case, for the CCl_4_+CLP model, we adapted the criteria in patients using previously published thresholds and cutoffs of organ function normality in mice,^17^,^18^,^19^,^20^ assuming values not meeting these criteria as indicators of organ impairment. For example, liver impairment was considered for bilirubin levels >0.6 mg/dL, renal impairment for creatinine levels higher than 0.5 mg/dL and brain impairment for brain water content higher than 80% and/or a NBT score >4. Impairment of the coagulation and circulatory systems were defined by an INR higher to 1.2 and renin concentrations higher than 1 µg/mL.^21^,^22^,^23^,^24^ Finally, mice were considered to have respiratory impairment when SpO_2_ was lower than 70%.^25^,^26^

The CCl_4_-CLP model bears some limitations. For example, although comparable to that described in the human disease, the model has a high mortality rate, which makes it difficult its use for the screening and testing of new drugs. However, the CLP model allows the fine-tuning of the severity of organ impairments and the mortality rate by using different gauge needles to puncture the cecum and by modifying the distance of the ligation to the ileocecal valve. Another aspect to consider is that the CLP model shows heterogeneity of organ impairments, which allows capturing the existing heterogeneity of the human disease, but it could hamper its reproducibility. Important to note that the CLP model, despite being a “gold standard” for sepsis research, has inherent limitations and does not fully replicate the complexity of human sepsis.^32^ It is likely that this limitation could explain why many treatment strategies that showed efficacy in the CLP model have failed in clinical trials. For the above-mentioned reasons, future refinements, including the implementation of the CLP model in female mice, are required.

In summary, the current study describes a new optimized experimental model of ACLF based on the combination of the well-known CCl_4_-induced chronic model of cirrhosis and the likewise widespread acute model of polymicrobial peritonitis induced by the ligation and puncture of the cecum (ie, CLP). The strongest asset of the CCl_4_+CLP model is that it faithfully reproduces the extrahepatic organ failures as well as the ramping systemic hyperinflammatory scenario present in patients with AD cirrhosis at risk of developing ACLF. Another major feature of the CCl_4_+CLP model is that short-term mortality is up to 30%, a similar mortality rate to that reported for ACLF patients. Importantly, the CCl_4_+CLP model was designed in mice, considering the extensive catalog of genetically engineered (ie, transgenic and knockout) possibilities existing in the mouse species, although it can be easily implanted in other rodent species such as the rat.

## Supplementary Material
