# Supplementary material for: An optimized peritonitis-induced ACLF model that reproduces the full spectrum of extrahepatic organ failures in mice
Source: Hepatol Commun. 2025 Jun 19;9(7):e0744. doi: 10.1097/HC9.0000000000000744 (PMC12180834; doi:10.1097/HC9.0000000000000744)
Supplement: Supplementary file 1 [file hc9-9-e0744-s001.pdf]

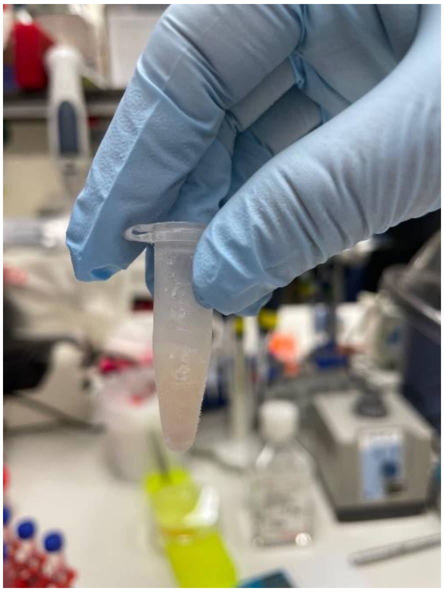

Supplementary Figure 1. Representative photograph of frozen ascitic fluid collected from the peritoneal cavity of a mice with CCl_4-induced cirrhosis.

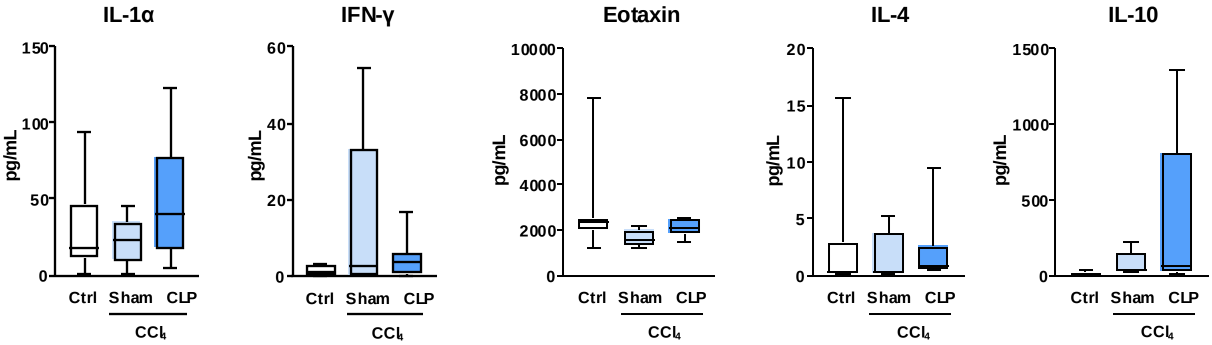

Supplementary Figure 2. Serum IL-1α, IFN-γ, Eotaxin, IL-4 and IL-10 protein levels determined by Milliplex technology. Results are expressed as box plots, the boxes show the interquartile range, the median values (horizontal lines) and the bars denote the highest and lowest values of the distribution.

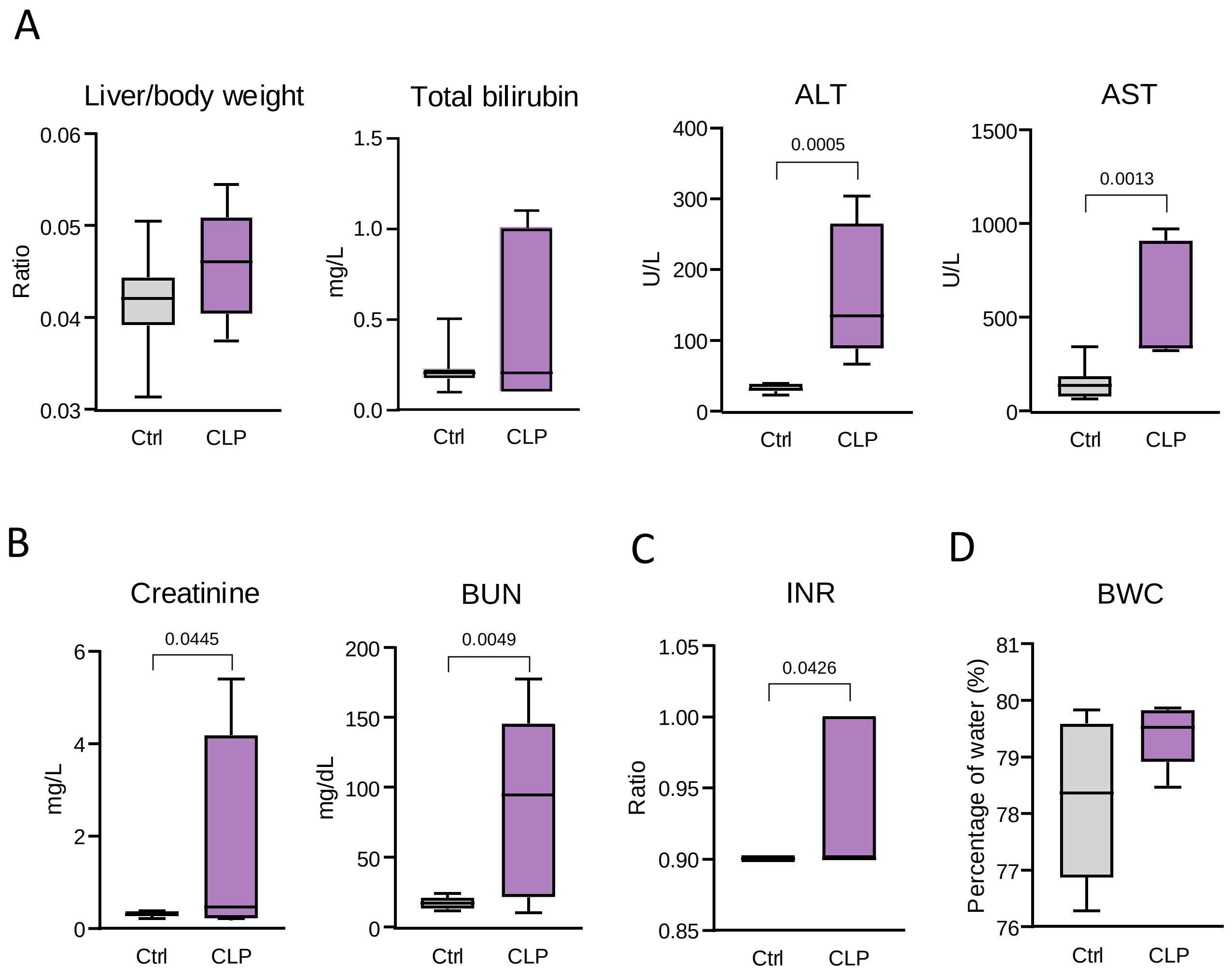

Supplementary Figure 3. Effects of CLP on mice without cirrhosis. (A) Liver weight, expressed as tissue-to-body weight ratio and serum bilirubin, alanine aminotransferase (ALT), and aspartate aminotransferase (AST) levels in healthy control mice (n=10) and healthy mice undergoing the CLP procedure (n=5). (B) Serum creatinine and blood urea nitrogen (BUN) levels in healthy control mice and healthy mice undergoing CLP. (C) International normalized ratio (INR) in control and CLP mice. (D) Brain water content (BWC) expressed as percentage of water in the tissue in control and CLP mice. Results are expressed as box plots, the boxes show the interquartile range, the median values (horizontal lines) and the bars denote the highest and lowest values of the distribution.

Study group	Liver	Kidney	Brain	Coagulation	Circulation	Respiration
**Control**	0/10 (0%)	0/10 (0%)	0/10 (0%)	0/10 (0%)	0/10 (0%)	0/10 (0%)
**CCl4+Sham**	1/10 (10%)	0/10 (0%)	0/10 (0%)	0/10 (0%)	0/10 (0%)	0/10 (0%)
**CCl4+CLP**	6/10 (60%)	6/10 (60%)	4/10 (40%)	4/10 (40%)	3/10 (30%)	5/10 (50%)

Supplementary Table 1. Prevalence of individual organ impairments in mice with CCl_4-induced cirrhosis undergoing CLP. Data are expressed as percentage of animals affected from each organ impairment within each study group.
